# Identification-While-Scanning of a Multi-Aircraft Formation Based on Sparse Recovery for Narrowband Radar

**DOI:** 10.3390/s16111972

**Published:** 2016-11-23

**Authors:** Yuan Jiang, Jia Xu, Shi-Bao Peng, Er-Ke Mao, Teng Long, Ying-Ning Peng

**Affiliations:** 1 School of Information and Electronics, Beijing Institute of Technology, Beijing 100081, China; jiangyuan@bit.edu.cn (Y.J.); maoerke@bit.edu.cn (E.-K.M.); longteng@bit.edu.cn (T.L.); 2 Department of Electronic Engineering, Tsinghua University, Beijing 100084, China; pengshibao@mail.tsinghua.edu.cn (S.-B.P.); ynpeng@mail.tsinghua.edu.cn (Y.-N.P.)

**Keywords:** narrowband radar, multi-aircraft formation (MAF), identification-while-scanning (IWS), sparse recovery, hierarchical basis pursuit (HBP)

## Abstract

It is known that the identification performance of a multi-aircraft formation (MAF) of narrowband radar mainly depends on the time on target (TOT). To realize the identification task in one rotated scan with limited TOT, the paper proposes a novel identification-while-scanning (IWS) method based on sparse recovery to maintain high rotating speed and super-resolution for MAF identification, simultaneously. First, a multiple chirp signal model is established for MAF in a single scan, where different aircraft may have different Doppler centers and Doppler rates. Second, based on the sparsity of MAF in the Doppler parameter space, a novel hierarchical basis pursuit (HBP) method is proposed to obtain satisfactory sparse recovery performance as well as high computational efficiency. Furthermore, the parameter estimation performance of the proposed IWS identification method is analyzed with respect to recovery condition, signal-to-noise ratio and TOT. It is shown that an MAF can be effectively identified via HBP with a TOT of only about one hundred microseconds for IWS applications. Finally, some numerical experiment results are provided to demonstrate the effectiveness of the proposed method based on both simulated and real measured data.

## 1. Introduction

Multi-aircraft formation (MAF) identification is an important task [1,2,3,4] for air search radar, where both the aircraft number and their motion information are of particular interest. For the narrowband radar, aircraft in the MAF can be densely distributed in a single beam and a single range cell of echoes. Although wideband waveforms have been introduced to distinguish between aircraft in difference range cells [4,5,6,7,8,9,10], a high A/D sampling frequency and huge data storage are required. Moreover, wideband waveforms may bring difficulties on target detection due to the energy being distributed along different range cells and the unknown range migration among different high range resolution profiles (HRRPs) [1]. Therefore, narrowband radar systems are still widely used in air search radars [1,11,12]. Though different aircraft in MAF may not be separated along the low range resolution profiles, different Doppler parameters, which are caused by different positions and motions of targets during a long time on target (TOT), provide a new approach for MAF identification [1,13,14]. In this regards, the third-order polynomial Fourier transform (PFT) [1] and many other time-frequency analysis (TFA) methods [13,14] have been proposed in a long TOT, e.g., one or several seconds. However, a long TOT cannot be obtained in many scenarios due to the following three reasons. First, the TOT is an important resource for radar, especially for wide-area search radar, and it needs to allocate the illumination time to detect targets from different directions. Second, modern radar is expected to have a quick response to environmental change, and the MAF information should be determined as soon as possible. Third, a dynamic frame-by-frame observation of MAF is expected in many applications to reflect the MAF time-varying motion. Specifically, for mechanically rotated or electronically scanned radar, it is hoped that an MAF can be identified in a single scan without decrease of the rotation speed as well as the data rate for target report. Therefore, the identification-while-scanning (IWS) in a short TOT is preferred for MAF identification in many practical applications.

The echoes of the MAF should be composed by different polynomial phase signals (PPS) with respect to different aircraft [1]. Furthermore, in this paper the echoes are derived as the composition of a multiple chirp signals (MCS) model in a short TOT of a single scan. Thus the IWS issue in a short TOT is equivalent to an estimation of multiple chirp components, in which the main problem is how to realize super-resolution of these components in a short TOT for IWS application. Fortunately, as the number of aircraft is limited, the MCS can be regarded as sparse in the whole parametric space. Therefore, in this paper the MAF identification is investigated by introducing sparse recovery (SR), which is recently discussed in radar area, especially for synthetic aperture radar (SAR) imaging [11,12,15,16,17,18,19,20]. The SR theory indicates that if a signal is sparse on some basis, it can be exactly reconstructed from limited samples with high probability. SR is originally realized by $l_{0}$ norm minimization, which is an NP-hard problem and intractable to solve directly. Fortunately, if the measurement matrix satisfies the restricted isometry property (RIP) [21] with appropriate restricted isometric constants, the $l_{0}$ norm minimization can be approximated to the solvable $l_{1}$ norm minimization, which can be realized by convex optimization or greedy algorithms. Compared with greedy algorithms, such as orthogonal matching pursuit (OMP) [22,23], convex optimization algorithms like basis pursuit (BP) [24] have higher recovery accuracy but much more computational complexity. In the MAF identification issue, convex optimization is preferred since the target number needs to be estimated accurately. On the other hand, off-grid problem of SR [25,26] may be inevitable since the real Doppler parameters of MAF can be any continuous real values while the grid of the basis are pre-discretized. Although refining the grids can increase the recovery probability, it will also create a heavy computation burden and recovery efficiency loss. Therefore, to solve the off-grid problem and maintain a moderate computation complexity, hierarchical recovery has been proposed in the field of SR. In literatures [27,28], a hierarchical matching pursuit is proposed and applied to sparse coding in image classification. In [29,30], grid refinement is utilized for better SAR and ISAR imaging. In these former works, though, the effective grid setting rule is not well discussed. In this paper, a hierarchical basis pursuit (HBP) method is proposed. It is clearly shown that the MAF can be effectively identified via HBP in a short TOT of only one hundred microseconds for IWS applications.

The remainder of the paper is organized as follows. In Section 2, the MAF signal model is established based on narrowband signals in a short TOT. In Section 3, the MAF identification is discussed by SR and the HBP method for IWS application is proposed with detailed performance analysis. In Section 4, some results of simulations and real measured data are provided to demonstrate the effectiveness of the proposed method. In Section 5, some conclusions are drawn.

## 2. Multi-Aircraft Formation Signal Model in Short Coherent Integrated Time

**Figure 1:**
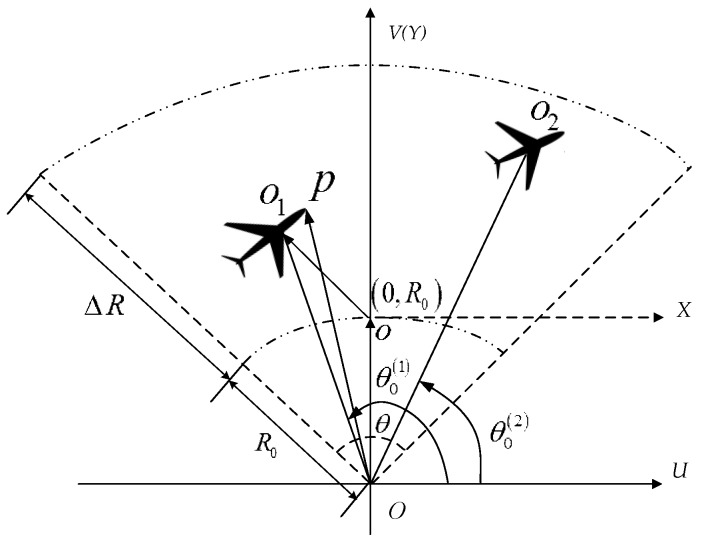
MAF geometry for the narrowband radar.

We have proposed a two-dimensional MAF signal model in [1] in long coherent integrated time (LCIT), e.g., several seconds. In this paper, we focus on the model for IWS applications in a short TOT, e.g., from several tens to one hundred micro-seconds, in a single scan for coherent integration. The geometry of an MAF scenario is rewritten in Figure 1 with two aircraft for simplicity. Three Cartesian coordinates are introduced for the convenience of derivation. *UOV* is the radar coordinate and the radar is located at the coordinate origin, *O*. *XOY* is the reference coordinate, where the axis *Y* coincides with the axis *V* and *X* is parallel to *U*. *|Oo| = R*_0_ and |·| is the absolute value operator. The third coordinate {*x*^(*k*)^*o*^(*k*)^*y*^(*k*)^*, k =* 1, ..., *K*} is the target coordinate, where *o*^(*k*)^ is the *k*-th aircraft geometry center, K is the aircraft number of the MAF and the origin of the *k*-th targets’ coordination is located at $\left(X_{0}^{\left(k\right)},Y_{0}^{\left(k\right)}\right)$ in *XOY*. The aircraft are assumed in the same beam and the same range cell from the constraint

(1) $$\left|X_{0}^{\left(k\right)}\right|\leq \left(R_{0}+\Delta R\right)\theta /2\begin{array}{cc} , & \;0\leq Y_{0}^{\left(k\right)} \end{array}\leq \Delta R$$

where θ is the azimuth beam width, ΔR is the range resolution of the narrowband radar. The radar light of sight (LOS) angle is $\theta_{0}^{\left(k\right)}$, corresponding to the angle $∠UOo^{\left(k\right)}$. The radial direction is denoted as the axis of *Y* and the tangential direction is the axis of *X*.

Normally, the motions of aircraft in the MAF cannot change rapidly for the safety reason, thus only the first and second order motions needs to be considered, i.e., the radical velocity $v_{y}^{\left(k\right)}$, the tangential velocity $v_{x}^{\left(k\right)}$, the radical acceleration $a_{y}^{\left(k\right)}$ and the tangential acceleration $a_{x}^{\left(k\right)}$, should be considered. Thus, an arbitrary scatterer *P* of the *k*-th target is located at ${\left(x_{p}^{\left(k\right)},y_{p}^{\left(k\right)}\right)}^{T}$in the *k*-th target coordinates, which corresponds to the location ${\left(X_{0}^{\left(k\right)}+x_{p}^{\left(k\right)},Y_{0}^{\left(k\right)}+y_{p}^{\left(k\right)}\right)}^{T}$ in radar coordinates and ${\left(\cdot \right)}^{T}$ is the transpose operator. The range from the scatterer *P* to the radar versus *t* can be expressed as

(2) $$r_{p}^{\left(k\right)}\left(t\right)=\left|OP\left(t\right)\right|=\left|Oo^{\left(k\right)}\left(t\right)+o^{\left(k\right)}P\left(t\right)\right|.$$

Usually, closely spaced scatterers cannot be effectively identified in a limited illumination time, even in several seconds of LCIT, so the phase differences among scatterers in a same aircraft can be omitted, which means that each aircraft in MAF contributes one Doppler component. Therefore, the MAF signal model can be approximated to the combination of *K* components of four-order polynomial phase signal (PPS) as

(3) $$s\left(t\right)=\sum_{k=1}^{K}\sigma^{\left(k\right)}\exp\left\{j2\pi \left[f_{1}^{\left(k\right)}t+f_{2}^{\left(k\right)}t^{2}+f_{3}^{\left(k\right)}t^{3}+f_{4}^{\left(k\right)}t^{4}\right]\right\},$$

where $f_{l}^{\left(k\right)}$ is the first order phase coefficient, l=1,2,3,4, which can be expressed as

(4) $$f_{1}^{\left(k\right)}=2\left(X_{0}^{\left(k\right)}\Omega^{k}\left(t\right)+v_{y,0}^{\left(k\right)}\right)/\lambda ,$$

(5) $$f_{2}^{\left(k\right)}=\left(a_{y}^{\left(k\right)}+\frac{{\left(v_{x,0}^{\left(k\right)}\right)}^{2}}{R_{0}}+\frac{X_{0}^{\left(k\right)}a_{x}^{\left(k\right)}}{R_{0}}\right)/\lambda ,$$

(6) $$f_{3}^{\left(k\right)}=\frac{v_{x,0}^{\left(k\right)}a_{x}^{\left(k\right)}}{\lambda R_{0}},$$

(7) $$f_{4}^{\left(k\right)}=\frac{{\left(a_{x}^{\left(k\right)}\right)}^{2}}{4\lambda R_{0}},$$

where $\sigma^{\left(k\right)}$ is the backscattering amplitude of the *k*-th target, $\Omega^{\left(k\right)}\left(t\right)$ is the equivalent rotation velocity generated by the *k*-th target’s tangential motion. Usually, the rotation velocity $\Omega^{\left(k\right)}\left(t\right)$ of each target can be assumed to be identical and denoted by Ω.

The linear phase coefficient $f_{1}^{\left(k\right)}$ causes the Doppler shift, while the quadratic term, cubic term and fourth-order term cause the Doppler dispersion. In the TOT, the Doppler frequency dispersion caused by the quadratic term, cubic term and fourth-order term are respectively denoted by $Fds_{2}$, $Fds_{3}$ and $Fds_{4}$ as

(8) $$Fds_{2}^{\left(k\right)}=2f_{2}^{\left(k\right)}T=2\left(a_{y}^{\left(k\right)}+\frac{{\left(v_{x,0}^{\left(k\right)}\right)}^{2}}{R_{0}}+\frac{X_{0}^{\left(k\right)}a_{x}^{\left(k\right)}}{R_{0}}\right)T/\lambda ,$$

(9) $$Fds_{3}^{\left(k\right)}=3f_{3}^{\left(k\right)}T^{2}=\frac{3v_{x,0}^{\left(k\right)}a_{x}^{\left(k\right)}}{\lambda R_{0}}T^{2},$$

(10) $$Fds_{4}^{\left(k\right)}=4f_{4}^{\left(k\right)}T^{3}=\frac{{\left(a_{x}^{\left(k\right)}\right)}^{2}}{\lambda R_{0}}T^{3}.$$

Normally, the velocity $v_{x,0}^{\left(k\right)}$ will be several hundred meters per second, the acceleration $a_{x}^{\left(k\right)}<10{\;m/s}^{2}$, and the radial range between the MAF and the radar $R_{0}>10\;\mathrm{km}$. For the application of IWS, the TOT T is about 100 ms. Also, the wavelength of far-range search radar will be larger than 3 cm, the Doppler frequency dispersion by the cubic and fourth-order term meets

(11) $$Fds_{3}^{\left(k\right)}<<\frac{1}{2T}=\frac{\rho_{f}}{2},$$

(12) $$Fds_{4}^{\left(k\right)}<<\frac{1}{2T}=\frac{\rho_{f}}{2},$$

where $\rho_{f}=1/T$ represents the Doppler center resolution cell. Equations (11) and (12) indicate that the dispersion $Fds_{4}$ and $Fds_{3}^{\left(k\right)}$ can be omitted in the short during time *T* as well as the cubic and fourth-order phase modulation. Moreover, the quadratic term $Fds_{2}^{\left(k\right)}$ will cause an obvious Doppler rate dispersion compared with $Fds_{4}^{\left(k\right)}$ and $Fds_{3}^{\left(k\right)}$. Thus, the MAF signal versus *t* for IWS application can be approximated as

(13) $$s\left(t\right)=\sum_{k=1}^{K}\sigma^{\left(k\right)}\exp\left\{j2\pi \left[f_{ak}t+f_{bk}t^{2}\right]\right\},$$

where the *k*-th aircraft is decided by its Doppler center *f_ak_* and Doppler rate *f_bk_*, jointly. Therefore, the narrowband coherent MAF signal in IWS application is equivalent to a model of *K* components of chirp signals, i.e., multiple chirp signal (MCS) model.

The 2D geometry *UOV* established in is an observation plane composed of the radar LOS and the instant velocity vector of MAF at *t* = 0. Actually, the targets of MAF may move out of the *UOV* plane in a real 3D space. Fortunately, in a short TOT, e.g., one hundred microseconds for IWS usage, the 3D motion can well be assumed bounded in the plane of *UOV* and the high-order motions can be omitted. Thus the MAF signal still can be modeled as the combination of different chirp components with respect to different aircraft in a 3D space. That is, the dimension of the matrices and the complexity of the recovery in a 3D geometry will be as same as those in 2D geometry in a short TOT.

Besides, for safety reasons, the aircraft in a stable MAF may normally keep the identical motion, which is called rigid MAF formation. In that case, the MCS can be verified to have the same Chirp rate [31]. However, in some scenarios, the aircraft in a MAF may have different motions and change their relative positions in the flight, which is called non-rigid formation. Also, two different MAF formations may possibly appear in the same range cell of the same beam. Therefore, this paper mainly discusses the non-rigid MAF identification as in Equation (13), and the rigid one can be regarded as a special case of Equation (13).

## 3. IWS MAF Identification via Sparse Recovery Methods

It is shown in Equation (13) that MAF identification can be realized for IWS application by estimating the number of chirp components as well as their parameters. However, the TOT in one IWS single scan is too short to discriminate each chirp component for conventional methods. Therefore, sparse recovery approaches are introduced in this Section to solve the resolution problem. Moreover, it is shown that the number of chirp components as well as their parameters, i.e., Doppler centers and Doppler rates, can be estimated by the sparse recovery, simultaneously. Based on Equation (13), the dictionary of sparse recovery consisted of the chirp atoms with different Doppler centers and rates for the over complete representation. However, retrieving the chirp information is a two-dimensional parameter search problem with a considerable computational burden. To reduce the computational complexity and sustain a high accuracy for the convex optimization, a HBP recovery method is further proposed for MAF identification in this section.

### 3.1. Overview of Sparse Recovery Theory

The framework of SR indicates that with the assumption of sparsity, the *N* × *1* dimensional signal vector **s** can be expressed as

(14) s=Ψf,

where **Ψ** is a *N* × *Z* dimensional basis matrix, **f** is a *Z* × 1 dimensional reconstructed signal vector with sparsity of *K*, i.e., only *K* elements of **f** are nonzero and *K* << *Z*. From the compressed sensing theory, the **s** can be recovered from its limited observation **y** with length of *M* and *M* << *N*, which can be expressed as

(15) y=Φs,

where **y** is the measurement vector and **Φ** is an *M* × *N* measurement matrix. Then we can obtain

(16) y=Φs=ΦΨf=Θf,

where the dictionary **Θ** = **ΦΨ**.

According to Equations (14)–(16), the recovery of **s** is equivalent to the recovery of **f**. Thus the following will only focus on the recovery of **f**. If the measurement matrix **Θ** is chosen properly, **f** will be recovered from the locations of the nonzero entries of **f**.

The principle of BP is to find a representation of the signal whose coefficients have minimal $l_{1}$ norm, as represented by Equation (17)

(17) $$\min{\left\|f\right\|}_{1}\;\mathrm{subject}\;\mathrm{to}\;\Theta f=y,$$

where **y** is the samples of the signal in a noise-free environment.

In the case of standard white Gaussian noise with deviation of σ, Basis Pursuit denoising (BPDN) is proposed, which is the solution of

(18) $$\underset{f}{\min}\left(\frac{1}{2}{\left\|y-\Theta f\right\|}_{2}^{2}+\lambda {\left\|f\right\|}_{1}\right).$$

Assuming the dictionary is normalized, then the parameter *λ* is related to the noise power, usually set as $\lambda_{C}=\sigma \sqrt{2\log(C)}$, with C is the cardinality of the dictionary.

BPDN is implemented by searching the best solution to Equation (18) on the whole **f** axis, which requires a large amount of time for computation. To make BPDN feasible in practical application, a HBP method is proposed in this paper by hierarchically search to reduce the computation burden remarkably.

### 3.2. MAF Identification by HBP Method

At first we prove the sparsity of the formation signal represented in the transform domain. Rewrite the formation signal in Equation (13) in the discrete form as

(19) $$s\left[n\right]=\sum_{k=1}^{K}\sigma^{\left(k\right)}\exp\left[j2\pi \left(f_{1}^{\left(k\right)}T_{s}n+f_{2}^{\left(k\right)}T_{s}^{2}n^{2}\right)\right],$$

and $s={\left(s\left[1\right],s\left[2\right],\cdots ,s\left[N\right]\right)}^{T}$. Form the basis matrix **Ψ** in a row vector consisted by different Doppler centers and Doppler rates as

(20) $$\Psi ={\left[\begin{array}{c} \exp\left(-j2\pi \left(p_{1}+q_{1}\right)\right)\\ \exp\left(-j2\pi \left(2p_{1}+2^{2}q_{1}\right)\right)\\ \vdots \\ \exp\left(-j2\pi \left(Np_{1}+N^{2}q_{1}\right)\right) \end{array}\begin{array}{c} \cdots \\ \cdots \\ \ddots \\ \cdots \end{array}\begin{array}{ccccc} \exp\left(-j2\pi \left(p_{1}+q_{Q}\right)\right) & \cdots & \exp\left(-j2\pi \left(p_{i}+q_{j}\right)\right) & \cdots & \exp\left(-j2\pi \left(p_{P}+q_{Q}\right)\right)\\ \exp\left(-j2\pi \left(2p_{1}+2^{2}q_{Q}\right)\right) & \cdots & \exp\left(-j2\pi \left(2p_{i}+2^{2}q_{j}\right)\right) & \cdots & \exp\left(-j2\pi \left(2p_{P}+2^{2}q_{Q}\right)\right)\\ \vdots & \ddots & \vdots & \ddots & \vdots \\ \exp\left(-j2\pi \left(Np_{1}+N^{2}q_{Q}\right)\right) & \cdots & \exp\left(-j2\pi \left(Np_{i}+N^{2}q_{j}\right)\right) & \cdots & \exp\left(-j2\pi \left(Np_{P}+N^{2}q_{Q}\right)\right) \end{array}\right]}_{N\times PQ},$$

where the subscript *i* in *p_i_* varies from 1 to *P*, and for each *i* the subscript *j* in *q_j_* traverses from 1 to *Q*. Thus there are *Ρ* × *Q* combinations for *p_i_* and *q_j_*, corresponding to each columns in **Ψ**.

Assume that only the first M samples of the formation signal *s*[*n*] can be obtained in one scan, i.e., $y={\left(s\left[1\right],\cdots ,s\left[M\right]\right)}^{T}$, which means the observation matrix **Φ** has the form as

(21) $$\Phi ={\left[\begin{array}{cccccccc} 1 & 0 & 0 & \cdots & 0 & 0 & \cdots & 0\\ 0 & 1 & 0 & \cdots & 0 & 0 & \cdots & 0\\ \vdots & \vdots & \vdots & \ddots & \vdots & \vdots & \ddots & \vdots \\ 0 & 0 & 0 & 0 & 1 & 0 & \cdots & 0 \end{array}\right]}_{M\times N}.$$

Then the dictionary **Θ** can be written as

(22) $$\Theta =\Phi \Psi ={\left[\begin{array}{ccccc} \exp\left(-j2\pi \left(p_{1}+q_{1}\right)\right) & \cdots & \exp\left(-j2\pi \left(p_{i}+q_{j}\right)\right) & \cdots & \exp\left(-j2\pi \left(p_{P}+q_{Q}\right)\right)\\ \exp\left(-j2\pi \left(2p_{1}+2^{2}q_{1}\right)\right) & \cdots & \exp\left(-j2\pi \left(2p_{i}+2^{2}q_{j}\right)\right) & \cdots & \exp\left(-j2\pi \left(2p_{P}+2^{2}q_{Q}\right)\right)\\ \vdots & \cdots & \vdots & \cdots & \vdots \\ \exp\left(-j2\pi \left(Mp_{1}+M^{2}q_{1}\right)\right) & \cdots & \exp\left(-j2\pi \left(Mp_{i}+M^{2}q_{j}\right)\right) & \cdots & \exp\left(-j2\pi \left(Mp_{P}+M^{2}q_{Q}\right)\right) \end{array}\right]}_{M\times PQ}.$$

Thus, the MAF samples **y** is represented by

(23) $$y_{M\times 1}=\Phi_{M\times N}s_{N\times 1}=\Phi_{M\times N}\Psi_{N\times PQ}f_{PQ\times 1}=\Theta_{M\times PQ}f_{PQ\times 1}.$$

The number of the nonzero elements of **f** in the basis matrix **Ψ** corresponds to the aircraft number, and the aircraft Doppler parameters can be estimated by the chirp basis corresponding to the nonzero elements in **f**. Specifically, the locations of the nonzero elements in **f** represent the aircraft’s Doppler center and Doppler rate, jointly. Therefore, **f** is sparse with only a few non-zero entries, which can be solved by BPDN in Equation (18).

Since the computation for the optimization of Equation (18) largely depends on the size of the measurement matrix **Θ**, the atoms of **Θ** can be designed in two hierarchies with different grids. Specifically, in the first hierarchy, the atoms in the measurement matrix **Θ**_1_, expressed as *θ*_1_(*p_i_, q_j_*), should have a wider range to ensure that the signal components can totally be covered. To keep **Θ**_1_ as small as possible, the grid among the atoms is allowed to be large to a great extent. While in the second hierarchy, atoms in the measurement matrix **Θ**_2_ have a much smaller grid, and only need to cover the small range of the non-zero atoms based on the sparse recovery result of the first hierarchy. Compared to the traditional BP method, the HBP method can keep the same accuracy and reduce the computational complexity significantly.

**Figure 2:**
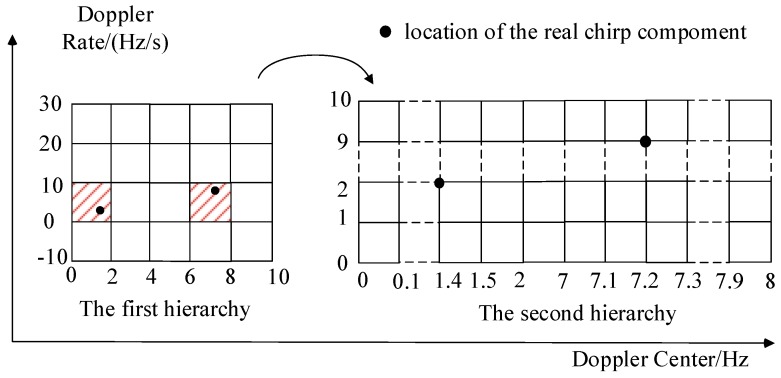
The illustration of the HBP strategy.

An example is given to demonstrate the implementation of the HBP strategy. The signal is supposed to be constructed by two chirp components with Doppler center $f_{a1}=1.4\;\mathrm{Hz},f_{a2}=7.2\;\mathrm{Hz}$ and Doppler rate $f_{b1}=2\;\mathrm{Hz}/s,f_{b2}=9\;\mathrm{Hz}/s$ respectively. The CPI is 100 ms. In the first hierarchy, the grids for the Doppler center and rate are set as 2 Hz and 10 Hz/s, as shown in Figure 2. In the second hierarchy, the dictionary is finer with more intensive grids of 0.1 Hz and 1 Hz/s. As shown in Figure 2 in the first hierarchy, the signal can be recovered by the items of the dictionary around the true value of the rough parameters. In the second hierarchy, the signal is recovered by the exact items as the Doppler parameters fall on the grid. The example above indicates that the tuning of the grid is important for the successfully recovery of the signal, which will be discussed in our future work.

### 3.3. The Discussion on Performance Analysis and the Grid Setting Rule for the HBP Method

To characterize the recovery ability of a given dictionary, the RIP and the coherence measure are two main metrics. Normally, the former is an NP-hard problem [19] to determine the RIP constants while the latter may be a simpler choice [32,33,34]. Although coherence measure is less accurate than RIP as the recovery condition, it has more explicit expression and is physically meaningful in our case of Equation (23). Therefore, in this section, the coherence is discussed and analyzed to test the recovery ability of the dictionary **Θ**. The coherence [17] of **Θ** is defined as the largest absolute inner product between any two columns *θ_i_*, *θ_j_* in **Θ**

(24) $$\mu \left(\Theta \right)=\underset{1\leq i<j\leq N}{\max}\frac{\left|\langle \theta_{i},\theta_{j}\rangle \right|}{{\left\|\theta_{i}\right\|}_{2}{\left\|\theta_{j}\right\|}_{2}}.$$

where 〈⋅,⋅〉 is the inner product operator. According to Theorem 12 of chapter 1 in [35], a sparse vector **f** can be successfully recovered by CS framework given a length-N observed echo **y** and the dictionary **Θ**, as long as the aircraft number *K* satisfies

(25) $$K<\frac{1}{2}\left(1+\frac{1}{\mu \left(\Theta \right)}\right).$$

According to the Welch bound [36], the coherence of a basis matrix is always in the range $\mu \left(\Theta \right)\in \left[\sqrt{\left(N-M\right)/\left(M(N-1)\right)},1\right]$. If N>>M, the lower bound for μ(Θ) approximates to $1/\sqrt{M}$. According to Equation (25), the upper bound for the aircraft number K which can be uniquely reconstructed will be $(1+\sqrt{M})/2$. The coherence of the atoms *θ_i_*, *θ_j_* in the measurement matrix **Θ** can be derived as

(26) $$\begin{array}{ll} \mu \left(\theta_{i},\theta_{j}\right) & =\frac{1}{M}\left|\sum_{n=1}^{M}\exp\left(-j2\pi \left(p_{i_{i}}T_{s}n+q_{i_{j}}T_{s}^{2}n^{2}\right)\right)\exp\left(j2\pi \left(p_{j_{i}}T_{s}n+q_{j_{j}}T_{s}^{2}n^{2}\right)\right)\right|\\ & =\frac{1}{M}\left|\sum_{n=1}^{M}\exp\left(-j2\pi \left(\left(p_{i_{i}}-p_{j_{i}}\right)T_{s}n+\left(q_{i_{j}}-q_{j_{j}}\right)T_{s}^{2}n^{2}\right)\right)\right|\\ & =\frac{1}{M}\left|\sum_{n=1}^{M}\exp\left(-j2\pi \left(\Delta pT_{s}n+\Delta qT_{s}^{2}n^{2}\right)\right)\right| \end{array},$$

where $\Delta p=p_{i_{i}}-p_{j_{i}}$ and $\Delta q=q_{i_{i}}-q_{j_{i}}$.

Notably, the coherence expression of Equation (26) has a similar expression to the ambiguity function [37], because they all reflect the correlation with the shifted parameters in a certain dimension.

The proposed sparse recovery method provides a super-resolution way to identify the aircraft. Since the theoretical resolution of the sparse recovery method depends on the dictionary’s atoms, the resolution can reach as high as possible if the selected steps of Δp and Δq are small enough, ideally. However, the possible resolution is limited by the coherence of the measurement matrix **Θ**. That is, if the steps of Δp and Δq are too small, the MAF signal cannot be reconstructed successfully since the coherences for the atoms in the dictionary are too large. Therefore, it can be inferred that the resolution for sparse recovery methods can be defined as the minimum steps for Δp and Δq meeting Equation (25) where *K* = 2. That is

(27) $$\rho_{p,\mathrm{CS}},\rho_{q,\mathrm{CS}}=\underset{\Delta_{p},\Delta_{q}}{\mathrm{argmin}}\left(\mu \left(\Theta \right)>\frac{1}{3}\right).$$

It should be mentioned that the recovery capability defined by Equation (25) is an issue of probability, which gives a strict condition to recover the signal with high recovery probability. However, in most cases which cannot satisfy Equation (25), the sparse recovery can still succeed if the SNR is high enough. In brief, much higher resolution can be obtained for the proposed HBP method than those of Equation (27) to satisfy MAF identification in one IWS scan.

Accordingly, the choice of the hierarchical grids is vital to the performance of sparse recovery. From analysis above, we give the grid setting rule for the MAF as follows. The atoms in the first layer can be rough in a wider range, with the grids in the same magnitude of the resolutions. Then the second hierarchy will reconstruct the signal with higher accuracy. The atoms in the second layer are selected in the neighbors of the recovery result in the first hierarchy with grids as small as one-tenth of the resolutions. It should be pointed that although smaller grids may bring errors for recovery, signals can still be identified well in relatively high SNR conditions. Moreover, in the second layer, the number of atoms is greatly reduced which can improve the recovery performance to some extent.

At last, the computational complexity is discussed. As BP method consists of a solution of the convex optimization problems and can be recast as a linear programming [24,38] if the non-zero components are real, which has the polynomial computation, i.e., typically $O(n^{3})$, where n is the number of atoms in the dictionary for sparse recovery. Thus it may be computational expensive or infeasible in real applications when n is large. In contrast, the greedy algorithms have been proposed to reduce the computation complexity to $O(nK^{2})$ with lower recovery accuracy, where *K* is the number of non-zero entries [38] and *K* << *n*. According to the proposed flowchart of HBP in Section 3, the HBP method reduces the computational complexity of BP [24] remarkably from $O(n^{3})$ to $O(n_{1}^{3})+O(n_{2}^{3})$, where $O(n_{1}^{3})$ is the computational complexity of the first hierarchy search and $n_{1}=n/C_{1}$ is the atom number in the dictionary of the first hierarchy and $C_{1}$ is the grid distance times of first hierarchy grid on the original dense grid. $O\left(n_{2}^{3}\right)$ is the second hierarchy search complexity and $n_{2}=C_{2}K$ is the atom number in dictionary of the second hierarch, where *K* is the sparsity, i.e., the aircraft number, and $C_{2}$ is the search atom numbers neighboring each of the *K* nonzero entries.

## 4. Numeric Experiments of the Proposed HBP Method

In this section, the results of both the simulation data and the real measured data are provided to demonstrate the effectiveness of the proposed HBP method for IWS applications. The system parameters are listed as follows. The signal carrier frequency $f_{c}=1\;\mathrm{GHz}$, the pulse repetition frequency (PRF) is 300 Hz, transmitting signal bandwidth B=1 MHz and the sampling frequency $f_{s}=2\;\mathrm{MHz}$. For a typical narrow-band coherent radar, assume that the antenna beam width is 4° with rotation speed 6 revolutions per minute (RPM), then the short TOT for coherent integration will be about 100 ms for the MAF identification in IWS applications.

### 4.1. MAF Performance Analysis

The aircraft number of the MAF is two and the slant range between the MAF and radar is 12 km. The locations in the reference coordinate are $X_{0}^{\left(1\right)}=-100\;m$ and $X_{0}^{\left(2\right)}=100\;m$ respectively. The motion parameters are listed in Table 1, from which the Doppler centers and Doppler rates for the two aircraft can be calculated from the parameters in Table 1.

**Figure 3:**
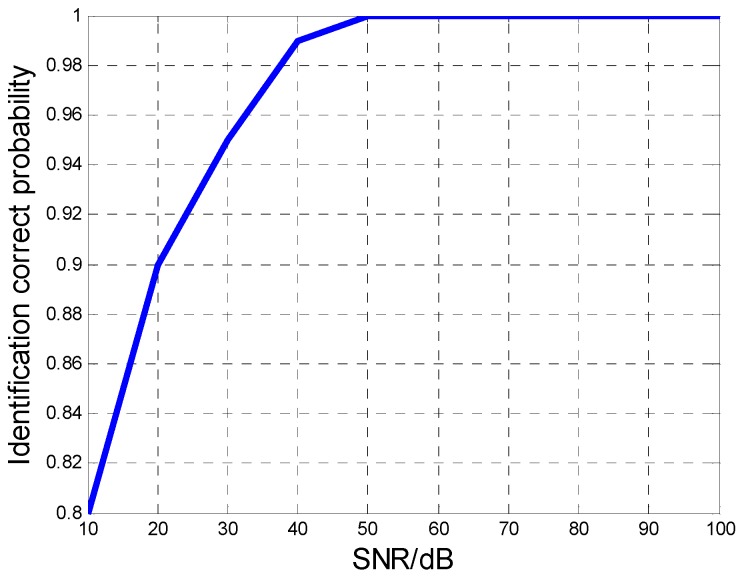
The identification correct probability of aircraft number versus SNR.

**Figure 4:**
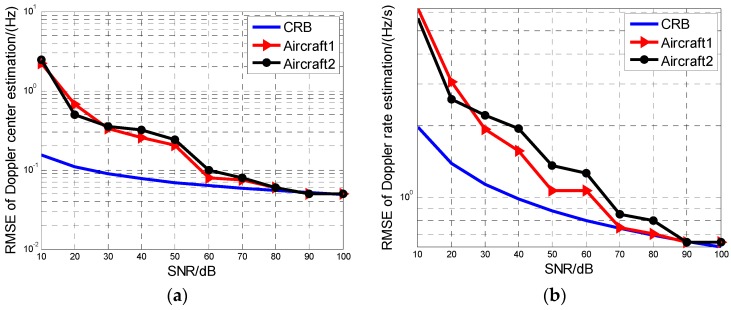
Estimation results of HBP compared to CRLB. (**a**) The Doppler center estimation; (**b**) Doppler rate estimation.

The MAF identification results by the HBP method are demonstrated as follows. Figure 3 shows the identification correct probability of the aircraft number versus SNR with 100 Monte Carlo samples, which illustrates that the correct probability reaches more than 90% when the SNR is higher than 30 dB. Figure 4a,b show the recovery accuracy for the Doppler center and the Doppler rate with 100 times of Monte Carlo simulation. The estimation accuracy of the proposed HBP method is compared with the CRLBs for each parameter. In Figure 4, both accuracies of the Doppler centers and Doppler rates all approaches to the CRLBs when SNR increases gradually, which shows that the proposed HBP method can obtain high accuracies.

**Figure 5:**
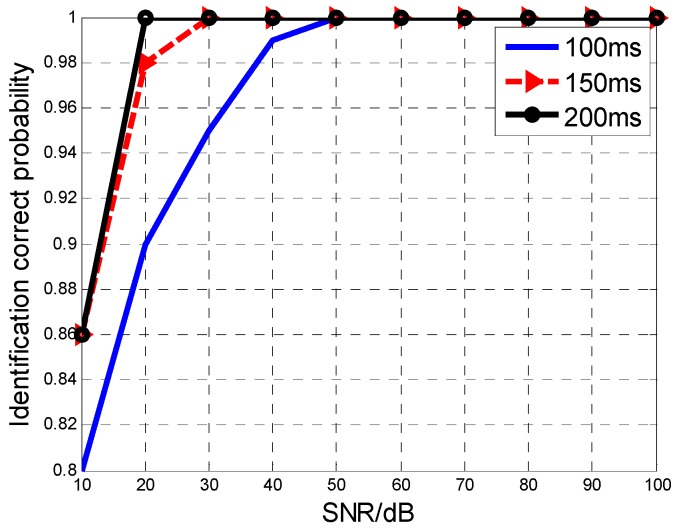
The MAF identification rate versus TOT.

With the increase of the TOT, the MAF identification will be more accurate. Figure 5 gives the correct identification probability of the aircraft number with different TOTs. When the TOT added up to 200 ms, the SNR requirement for 92% identification rate was reduced by nearly 15 dB.

**Figure 6:**
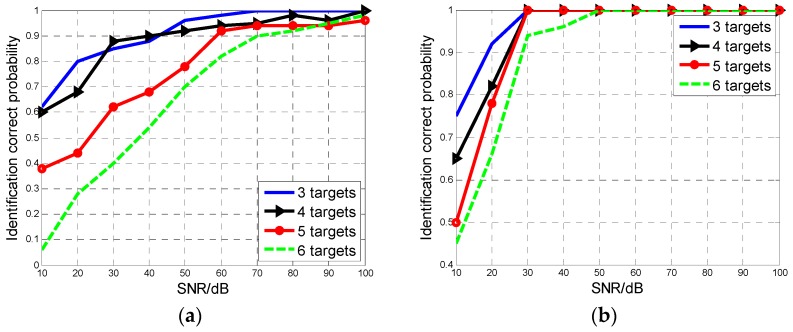
The correct identification probability versus MAF aircraft number. (**a**) *T* = 100 ms; (**b**) *T* = 200 ms.

The identification results for different aircraft numbers are shown in Figure 6a,b. For the TOT of 100 ms, the method can effectively identify as many as six targets with correct identification probability more than 80% when SNR is higher than 60 dB. For the TOT of 200 ms, MAF number can be correctly estimated with a probability of over 95% when SNR is higher than 30 dB.

### 4.2. Comparison with the PFT Method

**Figure 7:**
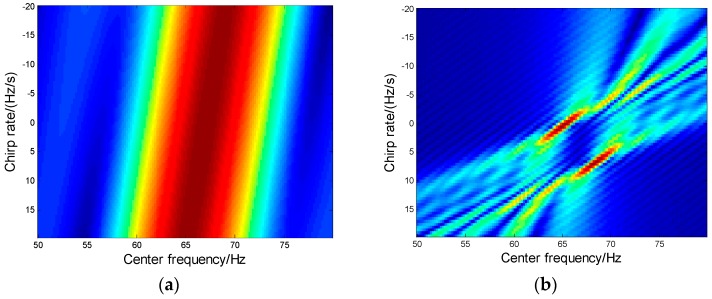
The second-order PFT result in $f_{2}-f_{1}$ plane. (**a**) Coherent time *T* = 0.1 s; (**b**) Coherent time *T* = 0.8 s.

In this part, HBP method is compared with the second-order PFT method proposed in [1], with the same MAF parameter of Table 1 with *T* = 0.1 s. The searching result for the second-order PFT method in $f_{2}-f_{1}$ plane is give in Figure 7, where the two aircraft cannot be identified due to the short TOT as well as the low parameter resolution. The result shows that the PFT method failed to estimate the MAF information. In fact, only when the coherent time *T* > 0.8 s can the parameters be well estimated by PFT. The results indicate that the proposed HBP is a super-resolution method while PFT is not suitable for IWS applications.

### 4.3. MAF Identification Based on Real Measured Data

The real measured data are obtained by an experimental radar system with parameters listed in Table 2. In the experiments, the location and velocity information can be obtained by the record of track for each aircraft, thus the real Doppler information can be calculated to evaluate the performance of the HBP method.

**Figure 8:**
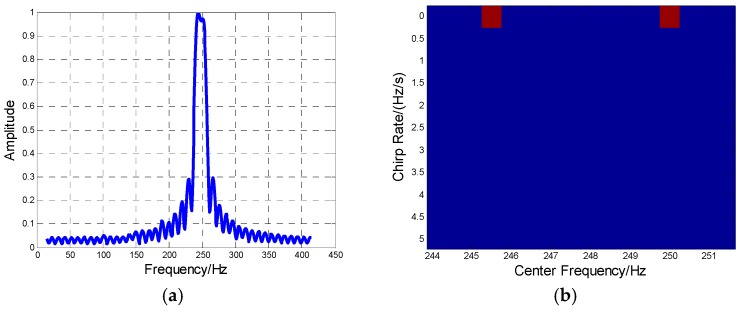
The two-aircraft MAF identification result. (**a**) Frequency spectrum of the MAF formation; (**b**) The MAF identification result by HBP.

**Case 1.** The MAF is constituted by two aircraft. The frequency spectrum of the signal is obtained by fast Fourier transform (FFT), as shown in Figure 8a. The identification result by HBP in one CPI is shown in the Doppler center–Doppler rate plane of Figure 8b, with the parameter estimation result in Table 3.

**Figure 9:**
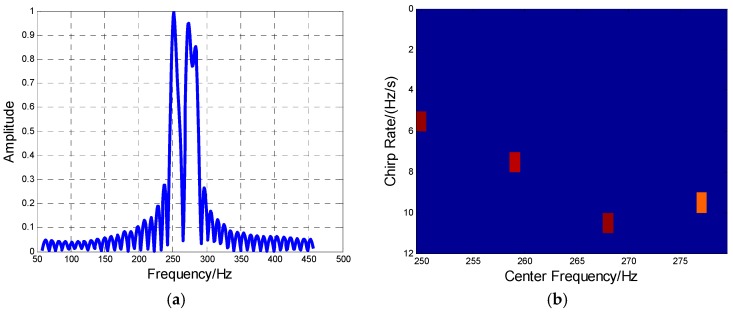
The four-aircraft MAF identification result. (**a**) Frequency spectrum of the MAF formation; (**b**) The MAF identification result by HBP.

**Case 2.** The data of MAF is formed by four aircraft. The frequency spectrum and recovery result are given as Figure 9a,b, where both aircraft number and their Doppler parameters are all well identified as seen in Table 4.

From the above experiment results, the effectiveness of the HBP for MAF identification is verified and the estimation accuracy for the Doppler center and Doppler rate is about 1 Hz and 2 Hz/s, respectively, which agrees with the simulation results in Section 4.1.

## 5. Conclusions

In this paper, MAF identification is discussed for IWS application for narrowband radar, based on a proposed MCS signal model where each aircraft can be represented by a chirp signal with a different Doppler center and Doppler rate. To realize super-resolution discrimination in a short TOT, sparse recovery is introduced for IWS applications in this paper. Furthermore, block-sparse MAF Doppler distribution is exploited to form an HBP method, which can reduce the computation complexity of the convex optimization algorithms and sustain high reconstruction accuracy, simultaneously. Thus the aircraft number as well as their Doppler parameters can be identified via the proposed HBP for each aircraft in MAF. Furthermore, the recovery condition, accuracy, resolution and computational complexity are discussed for the proposed method to show its high performance, respectively. Finally, some numerical experiment results of both simulated data and real measured data are provided to demonstrate the effectiveness of the proposed HBP method.

## Figures and Tables

**Table 1:** The motion parameters of the two-aircraft MAF.

Motion Parameters	Aircraft1		Aircraft2	
	Radical	Tangential	Radical	Tangential
Location	$Y_{0}^{\left(1\right)}=12,000\;m$	$X_{0}^{\left(1\right)}=-100\;m$	$Y_{0}^{\left(1\right)}=12,000\;m$	$X_{0}^{\left(2\right)}=100\;m$
Velocity	$v_{y}^{\left(1\right)}=100\;m/s$	$v_{x}^{\left(1\right)}=50\;m/s$	$v_{y}^{\left(2\right)}=100\;m/s$	$v_{x}^{\left(2\right)}=50\;m/s$
Acceleration	$a_{y}^{\left(1\right)}=0\;{m/s}^{2}$	$a_{x}^{\left(1\right)}=0.05\;{m/s}^{2}$	$a_{y}^{\left(2\right)}=1\;{m/s}^{2}$	$a_{x}^{\left(2\right)}=0.05\;{m/s}^{2}$

**Table 2:** System parameters in the real measured data experiment.

Fc	B	PRF	CPI	SNR
700 MHz	2 MHz	400 Hz	100 ms	40 dB

**Table 3:** Parameter estimation result.

	Aircraft1		Aircraft2	
	Doppler Center/(Hz)	Doppler Rate/(Hz/s)	Doppler Center/(Hz)	Doppler Rate/(Hz/s)
**Real value**	245.1	0.3	249.3	0.3
**Estimation value**	245.5	0	250.0	0

**Table 4:** Parameter estimation result.

	Aircraft1		Aircraft2		Aircraft3		Aircraft4	
	Doppler Center/(Hz)	Doppler Rate/(Hz/s)	Doppler Center/(Hz)	Doppler Rate/(Hz/s)	Doppler Center/(Hz)	Doppler Rate/(Hz/s)	Doppler Center/(Hz)	Doppler Rate/(Hz/s)
**Real value**	250.3	7.6	260.3	8.4	268.6	11.7	278.1	10.9
**Estimation value**	250.0	5.5	259.0	7.5	268.0	10.5	277.0	9.5
